# Clinical Effects of Mercury in Conservative Dentistry: A Systematic Review, Meta-Analysis, and Trial Sequential Analysis of Randomized Controlled Trials

**DOI:** 10.1155/2020/8857238

**Published:** 2020-08-12

**Authors:** Romeo Patini, Gianrico Spagnuolo, Federica Guglielmi, Edoardo Staderini, Michele Simeone, Andrea Camodeca, Patrizia Gallenzi

**Affiliations:** ^1^Department of Head, Neck and Sense Organs, School of Dentistry, Fondazione Policlinico Universitario A. Gemelli IRCCS, Università Cattolica del Sacro Cuore, 00168 Rome, Italy; ^2^Department of Neurosciences, Reproductive and Odontostomatological Sciences, University of Naples “Federico II”, 80125 Napoli, Italy; ^3^Institute of Dentistry, I. M. Sechenov First Moscow State Medical University, Moscow 119146, Russia

## Abstract

**Methods:**

A systematic literature search was conducted in four electronic databases (Ovid via PubMed, Web of Science, Scopus, and CENTRAL) including all available randomised controlled trials published in the last 15 years comparing the use of dental amalgam with composite resins in humans with a follow-up period of at least one year. The primary outcome was the Hg concentration in biological fluids (urine, hair, blood, and saliva) with the aim of assessing their reliability as biomarkers of Hg exposure. The risk of bias was assessed through the Cochrane Collaboration tool and the overall quality of evidence through the Grading of Recommendations, Assessment, Development and Evaluations (GRADE) system. The results of the meta-analysis were expressed using a random-effects model, and their power was assessed through the trial sequential analysis (TSA).

**Results:**

From the initial 2555 results, only 6 publications were included in the review: five were considered as having high risk of bias, whereas one as having moderate risk. Only two articles were eligible for quantitative analysis. The meta-analysis gathered data from 859 patients but was nevertheless not significant (*p* = 0.12). The TSA confirmed this evidence revealing that it was due to a lack of statistical power since the required information size (RIS) threshold is not reached.

**Conclusions:**

The existing evidence revealed that there are not enough data to support the hypothesis that restorations with dental amalgam can cause nephrotoxicity when compared with composite resins restorations.

## 1. Introduction

Dental caries is a progressive disease affecting the hard tissues of the tooth that originates from its surface and that could proceed until involving the dental pulp with an inflammatory process.

The aetiology of caries is multifactorial since several factors play a role in the onset and maintenance of the pathology and in its maintenance. In 1960, Keys [1] identified a triad of factors involved in the aetiology and pathogenesis of the disease: a specific bacterial flora, some predisposing factors of the host, and a diet rich in fermentable carbohydrates. Subsequent research showed that the exposure time of these factors also plays a crucial role [2].

The caries treatment involves removing the infected tissue and replacing it with biocompatible restoration material. Several materials can be used for filling cavities left by the removal of the infected tissues, but the most used are dental amalgam and composite resins.

Composite resins are a material, which guarantee a much better aesthetic result than dental amalgam. Many evidences, however, report that restorations made with composite resins do not have the same duration over time as those made in amalgam and they have a higher incidence of failures and relapses and higher costs and that the treatment's success is greatly influenced by the operator's experience [3–5]. Another very important aspect to consider is that, although numerous studies have been conducted on the biocompatibility of the constituents of composite resins on oral tissues, there is no evidence in the literature about the effects of composite resins on general health.

Dental amalgam is a liquid mercury and metal alloy mixture mainly used in dentistry to fill cavities produced by the treatment of dental caries. Low-copper amalgam commonly consists of mercury (50%), silver (∼22–32%), tin (∼14%), copper (∼8%), and other trace metals [6]. In the 1800s, amalgam became the dental restorative material of choice due to its low cost, ease of application, strength, and durability. In recent years, however, the use of dental amalgam has decreased considerably as some scientific evidence has revealed that amalgam vapours can be released during chewing and penetrate the systemic circulation by raising blood Hg levels above the threshold values [7]; other studies have correlated urinary Hg concentrations with possible nephrotoxicity and immune system pathologies [8–10]; more recent evidence, moreover, has focused on the hypothesis of neurotoxicity linked to the use of dental amalgam [11] or to the possibility of generating bacterial resistance to Hg and that these resistances can then transmit to other subjects through the exchange of oral fluids [12–15]. The studies that present critical issues with regard to amalgam, however, correlate the hypothetical adverse effects to the number of surfaces treated. In this regard, it should be noted that not all published studies are homogeneous as regards the number of filled surfaces (in some cases this information is not even reported) or that some do not involve an adequate follow-up period after exposure so that long-term effects are not visible.

In light of this, it is crucial to note that a recent systematic review that analysed studies published from 1996 to 2003 asserts that there is no evidence between amalgam and health problems [16]. Despite these very conflicting opinions, in July 2018, the European Union (EU) started a global expiring one year policy for reducing the use of amalgam for dental treatment of children under 15 years and of pregnant or breastfeeding women unless deemed strictly necessary by the dental practitioner on the ground of specific medical needs of the patient [17]. Such intervention can be considered a step forward to line up with the previous “Minamata Convention on Mercury,” an international treaty that aimed at protecting human health environment from emissions and releases of mercury and its compounds [18].

Therefore, it seems right to clarify and underline the most updated evidence on the subject as dental amalgam could remain the material of choice for the conservative treatment of enamel and dentin lesions in some categories of patients, such as special patient needs, in which a compliance that is essential for the success of caries treatment with composite resins can be achieved rarely.

According to the ongoing controversy over the safety of dental amalgam, the authors conducted a systematic review with meta-analysis and trial sequential analysis to investigate the effect of the exposure to Hg in adults and children with and without dental amalgam fillings measuring the Hg concentration in various biological fluids (urine, hair, blood, and saliva) in order to assess their reliability as biomarkers of Hg exposure from dental amalgam fillings. Also neurological and social-behavioural effects were evaluated as secondary outcomes.

## 2. Materials and Methods

### 2.1. Protocol Development and Eligibility Criteria

The authors designed a detailed protocol following the Preferred Reporting Items for Systematic Reviews and Meta-Analyses statement [19]. Following the PICO format, a focused question was also developed: “Can the use of dental amalgam in restorative dentistry in children or adults cause neurotoxicity, nephrotoxicity, or an increase in mercury percentage in blood when compared with composite resin?”

### 2.2. Search Strategy

A comprehensive systematic literature search was performed in four databases (Ovid via PubMed, Web of Science, Scopus, and CENTRAL) by two calibrated examiners (FG and AC). All available randomized controlled trials (RCT) published from January 1995 to March 2020 conducted on humans were selected. No language restrictions were applied.

Search strategy comprehended a combination of free text words and MeSH terms reported as follows:

(“dental amalgam”[Title/Abstract/MeSH]) AND (“gingival crevicular fluid” OR “health status” OR “mercury” OR “mercury poisoning” OR “lichen planus” OR “lichenoid eruptions” OR “mouth diseases” OR “mouth mucosa” OR “wound healing” OR “xerostomia” OR “corrosion” OR “craniomandibular disorders” OR “patient satisfaction” OR “hypersensitivity”[Title/Abstract/MeSH]).

The search strategy reported above was designed for MEDLINE PubMed and then adapted to the other three databases. A manual search was conducted on *European Journal of Oral Sciences, Journal of Oral Pathology and Medicine, British Dental Journal, Clinical Oral Investigations, Gerodontology, Journal of Dental Research,* and *Dental Materials* analysing all available RCTs published between January 1995 and March 2020.

The bibliographies of all articles included were consulted with the aim of analysing as many articles as possible.

### 2.3. Selection Criteria

Since RCTs are the studies that give the strongest scientific evidence, the authors decided to include all available RCTs published between January 1995 and March 2020, conducted on adults or children comparing the use of dental amalgam with composite resins. With the aim of highlighting any relationship between dental amalgam and neurotoxicity and nephrotoxicity, the authors defined as primary outcome the Hg concentration in various biological fluids (urine, hair, blood, and saliva). Social-behavioural effects were evaluated as secondary outcomes.

### 2.4. Exclusion Criteria

1. Case report, case series, any type of observational studies, letters, and narrative or systematic reviews
2. Studies published before January 1995
3. Grey literature
4. *In vitro* studies
5. Animal studies
6. Studies conducted on nonhealthy subjects in the enrolment phase
7. Studies with less than 1 year of follow-up

### 2.5. Selection of Studies

Two authors (FG and AC) dealt with the screening of the studies independently and in duplicate. Special designed data extraction forms were used for this purpose. An author supervisor (RP) was consulted in case of disagreement. The first step of the screening process was conducted on title and abstract; if the information included in these sections were not sufficient to make a decision, the full report was obtained for further screening. Agreement level between reviewers was evaluated through Cohen's kappa coefficient (*k*).

The evaluation of the methodological quality of the included studies was performed through the use of the Cochrane Collaboration's tool for assessing risk of bias in randomised trials. An adjunctive analysis was performed independently by two reviewers (FG and AC) regarding the overall quality of evidence at the outcome level using the Grading of Recommendations, Assessment, Development and Evaluations (GRADE) system.

### 2.6. Assessment of Heterogeneity

Review Manager (RevMan) software was used for the assessment of heterogeneity of the studies included in the meta-analysis [20]. The compatibility of the observed differences across the results with chance alone was calculated using the chi-square test and the *I*^2^ test. In case of *p* value < 0.1, heterogeneity was considered significant. The *I*^2^ test describing the heterogeneity-linked percentage of total variation across studies was considered as measure of heterogeneity, following the subsequent scheme:

- 0–40%: might not be important
- 30–60%: may represent moderate heterogeneity
- 50–90%: may represent substantial heterogeneity
- 75–100%: considerable heterogeneity [21]

### 2.7. Data Synthesis

After careful evaluation of all the selected full-text, only six RCT were included in this review. Even if the majority of the abovementioned studies were homogeneous in terms of study population demographics, the primary outcome of the present review was not evaluated in all of them. For this reason, a quantitative analysis was possible only for the data reported homogenously in at least two studies. The meta-analysis was conducted with a fixed-effects model comparing mean differences and standard deviations in case of continuous data. In case of a not-negligible heterogeneity (>50%) among studies, a random-effects model was used. The meta-analysis results underwent an adjunctive analysis with the aim of correcting them for the presence of alpha and beta errors and assessing the power of the analysis; for this scope, the authors used the trial sequential analysis (TSA) software (version 0.9 beta, http://www.ctu.dk/tsa). TSA software gave the possibility to calculate the required information size (RIS), the alpha-spending function, the trial sequential monitoring boundaries for benefits and harms, and the futility boundaries. So all data of the single trials were entered into the TSA software; the alpha error was set at 0.05 and the beta error at 20%. A correction for heterogeneity was performed according to the results of the meta-analysis. Trials having at least three domains assessed at high or unclear risk of bias were defined as at high risk, and trials with less than three domains assessed at high or unclear risk of bias were considered as having low risk. The results of the TSA analysis are presented as a graph with a cumulative z-curve and its relationship with the other curves (trial sequential monitoring boundary, the futility boundary, and the RIS threshold).

## 3. Results

### 3.1. Results of the Search

A systematic electronic search was performed involving four databases: MEDLINE (via PubMed), CENTRAL, Scopus, and Web of Science. The abovementioned databases retrieved 895, 97, 1234 and 329 results, respectively, for a total of 2555. Two reviewers (AC and FG) independently and in duplicate screened 2308 articles as results of the elimination of 247 duplicates. Title and abstract analysis led to the elimination of 2293 articles, so 15 results were selected for full-text analysis. Nine studies were excluded after full-text evaluation; their references are listed in the excluded studies table along with rationale for exclusion (Table 1).

**Figure 1:**
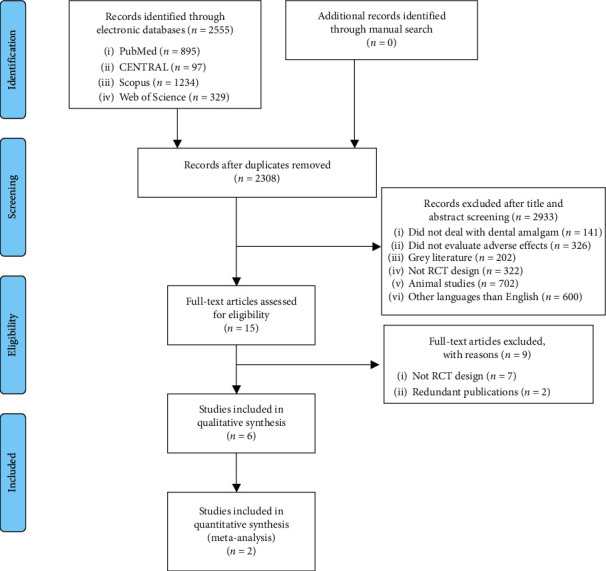
Flow chart of the search strategy.

Six studies were included in the review (Figure 1).

No additional publications were found through the manual search or in the bibliographies of the included studies. Cohen's kappa value for global interreviewer agreement was excellent, being 0.82 ± 0.12.

### 3.2. Included Studies

This review included six studies. Trials took place in USA, Portugal, and Germany giving birth to three, two, and one publications, respectively. All trials had a parallel group design, except for Halbach et al. that included also a third untreated group [33]. All dental examinations and procedures took place only in university dental clinics [32–34] or also in community-based ones [24, 35, 36]. Five studies enrolled a population of children (between 6 and 12 years), whereas only one enrolled adult people (20–50 years). The articles reported data about the Hg concentration in urine, hair, and blood [32, 33], and some reported neurological and social-behavioural effects of amalgam restorations [34, 35], whereas Bellinger et al. reported data belonging to the two areas [24].

Study characteristics are reported in Table 2.

### 3.3. Characteristics of Participants and Interventions

Peadiatric participants were enrolled if they needed a conservative caries treatment; they were divided in two treatment arms: dental amalgam arm if the lesion was restored with amalgam and composite resin arm if restoration was carried out with composite resin material. All children belonged to the New England Children's Amalgam Trial (NECAT) and to the Casa Pia School System Trial (CPSST).

Children were included if they had no previous amalgam restorations, if they had at least two dental caries, both located in posterior teeth including occlusal surfaces, and in absence of physician-diagnosed psychological, behavioural, neurological, immunosuppressive, or renal disease.

Randomization of children was performed with a stratification following their geographical origin or the school they attended.

For children belonging to the CPSST, the urinary mercury analyses were performed with continuous cold-flow, cold-vapour atomic spectrofluorometry and a PSA Merlin mercury analysis (Questron Corp, Mercerville, NJ) [32, 34]. CPSST's children were also evaluated about the presence of neurological hard signs (NHSs) and soft signs (NSSs). NHSs are considered predictive of damage to specific neural structures; on the other hand, NSSs are rather predictive of central nervous system dysfunctions. Tremor was recorded apart since it is one of the most frequent manifestations of mercury toxicity. The NHSs and NSSs analyses were performed by two neurologists once a year for 7 years starting from the baseline for NHSs and starting from year 2 for NSSs. Neurological examination was performed according to standard practice criteria [37, 38].

Urinary mercury was measured also in children belonging to the NECAT with the help of an immunochemical nephelometric method from Beckman Coulter (Fullerton, Calif). The unit of measures were the same reported in CPSST. The same analytic method as per urinary mercury was also used for the analysis of mercury deposits in hair. Publications showing data from the NECAT presented also data on full-scale IQ (according to the Wechsler Intelligence Scale for Children, Third Edition, WISC-III), on visuomotor ability assessment index and the general memory index (gathered from the Wide Range Assessment of Visual Motor Ability and from the Wide Range Assessment of Memory and Learning, respectively). The WISC-III was administered 3 times: at baseline prior to caries restoration and 3 and 5 years after baseline. The Wide Range Assessment of Visual Motor Ability and the Wide Range Assessment of Memory and Learning were administered twice: at the baseline and after 4 years. In NECAT also the social-behavioural outcomes contained into the Child Behaviour Checklist (CBCL) were analysed [39]. Such checklist was administered to a parent at baseline prior to dental treatment and 5 years later, at the completion of the trial. The main areas analysed by the checklist were: competence, internalizing behaviour problems, externalizing behaviour problems, and total problem behaviours. In the article by Shenker et al., reporting analysis of data gathered from a subgroup of children enrolled in the NECAT, immunological parameters were evaluated: white blood cell enumeration, assessment of T- and B-cell responsiveness, and analysis of neutrophil and monocyte responsiveness. Total WBC enumeration and distribution of immune cells were performed through the haemocytometer and flow cytometer. Analyses were carried out at baseline (patients enrolment), 7 days, and 6, 12, and 60 months after the enrolment [36].

Since only values regarding the urinary levels of mercury were homogenously reported in publications showing data from NECAT and CPSST, the authors decided to carry out a quantitative analysis only regarding that data. Qualitative analysis was carried out about the other data.

Adult population considered in the present review was the one presented by the RCT of Halbach et al. [33]. Patients were included if they suspected that dental amalgam was affecting their health status. Inclusion criteria were absence of any prosthetic rehabilitation or unsuccessful endodontic treatment and general health status. Patients were thereby excluded if they reported any type of physical illness or mental disorder. Randomization of patients was performed stratifying them according to the total number of tooth surfaces filled with amalgam (1–12, 13–18, and 19–25 surfaces) within each group. Patients were divided in three arms: A—removal of dental amalgam and substitution with composite resin; B—removal of dental amalgam, detoxification procedure, and substitution with composite resin; C—no removal of dental amalgam. Only patients belonging to arms A and C were considered in the review. The authors investigated concentrations of total, inorganic, and organic Hg in red blood cells and plasma and mercury concentration in morning urine. Such measurements were conducted at the time of prescreening and after randomization into groups. Subsequent analyses took place for the first time in the dental session in which amalgam was removed (for group A) or in the first dental check-up after randomization (for group C); then, other samples were taken at days 60, 360, and 540 and additionally at days 1, 3, 9, and 30 in group A. Additional urine samples were collected in day 180 from patients belonging to group A. Mercury concentration was determined through cold vapour atomic absorption spectrometry with a gold trap (Hg-Mess-2–87, Leunawerke, Leuna, Germany).

### 3.4. Risk of Bias in Included Studies

**Figure 2:**
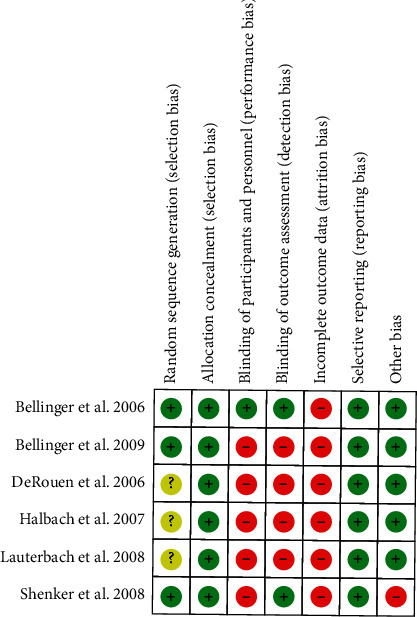
Risk of bias summary across all included studies.

**Figure 3:**
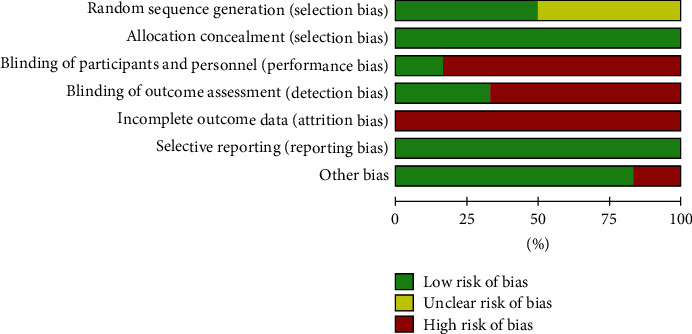
Risk of bias graph with overall percentages of bias for each domain.

The evaluation of the risk of bias of the included studies is summarized in Figures 2 and 3.

Such evaluation was conducted using the Cochrane collaboration tool for assessing the risk of bias. Because of the different appearance of the materials to be evaluated in the trials, participants could not be blinded; for this reason, the reviewers decided to exclude participants' blindness from the judgment regarding the performance bias.

The methodological quality of the included studies was moderate for one study [24] and low for the other 5 studies [32–36]. The shortcomings mostly concerned domains 3, 4, and 5 (blinding of personnel, outcome assessors, and incomplete outcome data) because of the lack of information regarding the blinding of medical and laboratory staff and the high rate of dropouts.

Methodological quality of trials was also analysed for the purposes of the TSA, and one study was considered as at low risk of bias [24], while the other one included in the analysis was considered as at high risk [32].

The GRADE system gave information regarding the certainty of the conclusions and strength of the evidence (Table 3).

Even if the meta-analysis has drawn conclusions from RCT that should be considered as the best evidence in scientific literature, data regarding the level of urinary mercury in children after 5 years of restoring dental caries with dental amalgam or composite resin were considered to have only moderate strength of evidence because of the high heterogeneity among studies and the presence of one study assessed as having high risk of bias.

### 3.5. Effects of Interventions

Three publications, among the six included in the present review, presented the results of a RCT known as NECAT and conducted between September 1997 and March 2005 in five community health dental clinics in Boston (Mass) and one in Farmington (Me). Each publication investigated and gave results about different aspects of the health of children whose caries were restored using either dental amalgam or mercury-free composite materials.

Bellinger et al. in 2006 presented data regarding mercury levels in urine and hair and about three neuropsychological outcomes (WISC-III full-scale IQ, general memory index, and visuomotor composite) in 534 children [24]. The level of urinary mercury was measured five years after the enrolment; the authors found that patients belonging to the amalgam group had a significantly higher level than children whose caries were restored with composite resin (0.9 *μ*g/g vs. 0.6 *μ*g/g; *p* < 0.001). On the contrary, concentration of mercury in hair was found to be similar between groups (0.4 *μ*g/g vs. 0.5 *μ*g/g). All the neuropsychological outcomes assessed 4-5 years after enrolment reported an increase in both groups without any statistical difference between them. Anyway, all the scores of the amalgam group increased more than the ones of the resin composite group. The authors stated a significant dropout rate during the study. Eighty-three patients were lost before the assessment of the neuropsychological outcomes and additional 42 patients before the mercury urinary check-up (39 and 20 for the amalgam group and 44 and 22 for the composite group, respectively).

In 2008, another research group published data about the psychosocial status of children enrolled in the NECAT [35]. Children were evaluated comparing data at baseline and 5 years later. Among the four main scales of the CBCL, a significant improvement was noted in the amalgam group with respect to the composite group on the domain “Total Problem Behaviour” (*p* < 0.007), and a weaker but still significant improvement was noted for the amalgam group in the “Internalizing” domain (*p* < 0.03). Even in the subscales, patients belonging to the amalgam group demonstrated better improvements than the nonamalgam group patients. Anyway, statistical significance was achieved only in three domains: “Delinquent Behaviours,” “Activities,” and “Anxious/Depressed,” with different grade (*p* < 0.002, *p* < 0.03 and *p* < 0.04, respectively).

The last included study that presented data drawn from the NECAT was published by McKinlay et al. in 2008 [36]. The authors evaluated immunological parameters at baseline (patients enrolment), 7 days, and 6, 12, and 60 months after the enrolment. A fluctuation of lymphocytes, monocytes, and neutrophils was observed but without statistically significant differences. Results about functionality of T-cells and monocytes revealed a decline at 5–7 days after treatment if compared with the composite group, even if not significant. On the contrary, no differences were detected at subsequent time points (6, 12, or 60 months). B-cells functionality demonstrated no differences between the various time points and the two groups. Neutrophils exhibited fluctuations in both treatment groups and among the various time points but without any type of significance neither among groups nor the time points.

Two publications reported data gathered from the CPSST. This was a 7-year trial starting in January 1997, which enrolled children aged 8–10 years during the recruitment phase. Participants were chosen among children attending 7 different campuses in the city of Lisbon. The publications investigated eventual nephrotoxicity or neurotoxicity of dental amalgam restorations in children.

In 2006, DeRouen et al. randomized 507 children and measured the urinary mercury levels every year for 7 years [32]. The authors declared a significant dropout rate since 55 patients did not complete the study through the first 5 years, and additional 96 were lost in the last two years. Anyway, starting from the first year of experimentation, mercury urinary levels were found to be significantly higher in the amalgam group (*p* < 0.001). Difference between groups was around 1.5 *μ*g/g in the first 3 years of follow-up, and then, it declined to around 1.0 *μ*g/g in the subsequent years.

Lauterbach et al. in 2008 investigated some neurological parameters in the population that made up the sample of the CPSST [34]. Such parameters were evaluated every year during the entire period of the trial even if only the 55% of the children originally enrolled completed the study. The analysis of NHSs and positional tremor revealed differences between the groups and among the years, but they never reached statistical significance; furthermore, not even the trend of modifications seemed to be consistent since higher NHSs rate in the amalgam group was seen only in the 1^st^, 2^nd^, 4^th^, and 6^th^ year time points. The trend of the NSSs followed the one of the NHSs except for the 2^nd^ year of observation; in fact, in that time point, children whose caries were restored with composite resin demonstrated a statistically significant higher rate of NSSs (more than 10% with *p* = 0.02) with respect to the dental amalgam group.

The only study included in the review that dealt about an adult population was written by Halbach et al. in 2007 [33]. The enrolment phase took place between April 1998 and July 2002. The authors declared that 91 patients composed the initial population, but at the end of the trial, such number was reduced to 74 because of dropouts. The study lasted approximately 18 months during which in each of the groups analysed, it was noted a decline of the total mercury levels in erythrocytes and then an early in the composite resin group or late increase in the dental amalgam group. The total mercury concentrations in plasma demonstrated an initial decline until they reached a steady-state level in the composite group, while they continued to decrease in the amalgam group. In both plasma and red cells, the levels of inorganic mercury showed a very similar trend: in the composite group, the levels decreased until reaching a steady-state (around day 60), while in the amalgam group, they remained constant. Only little and not significant deviations were observed in organic mercury concentrations of plasma over the whole study period in both groups. The same concentrations in red cells, on the contrary, raised in the composite group at the first time point (day 60), while in the amalgam group, they diminished slowly in the first year of observation until reaching the baseline level at the end of the study period. No statistically significant differences were detected regarding urinary mercury.

The quantitative analysis was conducted only on the unique outcome homogenously reported at least on two trials: urinary mercury concentration in children 5 years after restoration of dental caries with dental amalgam or composite resin.

**Figure 4:**
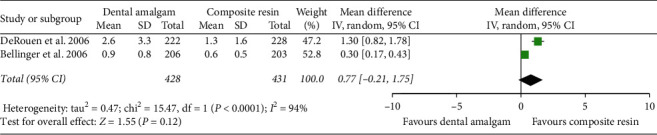
Forest plot of comparison for urinary mercury concentration after 5 years of exposure to dental amalgam or composite resin in children.

The meta-analysis of the two included trials (one assessed as having high risk of bias and the other one as having moderate risk) analysed data of 859 patients and did not find evidence to determine that dental amalgam restorations in children increased the urinary mercury levels after 5 years of observation (mean difference: 0.77 *μ*g/g; 95% CI: −0.21 to 1.75 (*p* value: 0.12), heterogeneity: Chi^2^ = 15.47, df = 1 (*p* value: < 0.0001); *I*^2^: 94%) (Figure 4).

**Figure 5:**
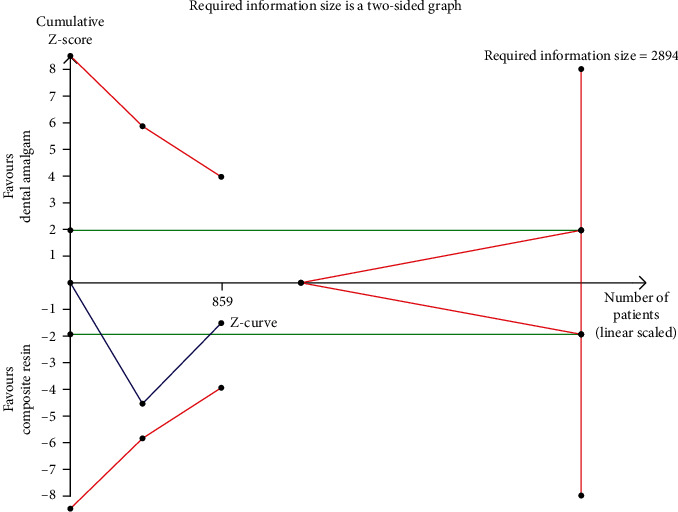
Trial sequential analysis for urinary mercury concentration after 5 years of exposure to dental amalgam or composite resin in children.

Even if the result of the meta-analysis was found not to be significant, the adjunctive TSA revealed that such nonsignificance was not due to a hypothetical equivalency between the two interventions but rather to a lack of statistical power. In fact, the TSA cumulative z-curve did not cross the alpha-spending function and the conventional boundaries; moreover, it did not reach the RIS threshold (Figure 5).

## 4. Discussion

The meta-analysis and the trial sequential analysis conducted on the selected RCTs revealed that there are not enough data to support the hypothesis that caries restorations with dental amalgam can cause a statistically significant increase in urinary mercury levels in children when compared with composite resins restorations. The authors decided to conduct the systematic review by including only RCTs since they are considered as having high strength of evidence. However, in the literature, there are numerous observational studies and case reports that detected a high amount of side effects related to the use of dental amalgam. One of the most investigated topics was antibiotic resistance. In fact, Wireman et al. in 1997 reported that some antibiotic resistant bacteria could also be mercury-resistant [40]; it has been, moreover, considered that genes linked with antibiotic resistance are susceptible to be transferred [41]. So, observational studies have been conducted with the aim of investigating the possible association between dental amalgam and the developing of antibiotic resistance in oral cavity bacteria. Nevertheless, the results of such investigations gave controversial results [15, 42]. Another relevant topic investigated in children was the eventual change in urinary porphyrin excretion exerted by mercury. In fact, as demonstrated by Bowers et al. in 1992, mercury could interfere with heme synthesis, thus causing the presence of urinary coproporphyrin [43]. Starting from this evidence, Geier et al. conducted a further analysis from the CPSST, in which they found an around 5–10% increase of mercury-associated porphyrins in subjects belonging to the dental amalgam group when compared with children whose caries were filled with composite resin [44]. Observational studies conducted on adult populations mainly focused on three big areas: mental disorders, hypersensitivity, and lichenoid lesions and perinatal medicine. The increase of mercury concentrations in breast milk, umbilical cord, and amniotic fluid was found to be statistically significant in the majority of publication reports about this topic [45–49], whereas controversial results have been published regarding the hypothetical influence of dental amalgam on the onset of mental disorders (mainly Parkinson and Alzheimer disease) [50–52] and of lichen planus and associated lesions [53–55]. Indeed, most of the evidence supporting the thesis of a link between oral mucosal reactions and dental amalgam are based on case reports [56–62]. Moreover, it has to be reported that some authors published cases of burning mouth syndrome and orofacial granulomatosis arisen in patients previously treated with dental amalgam [63, 64].

The qualitative analysis of studies included in the present review revealed a general and transversal lack of evidence towards the potential adverse and toxic effects of amalgam; this could have clinical implications in daily dental practice and induce to resize the policies implemented by various states of the EU deliberately against the use of amalgam in dentistry. In fact, after the European Mercury Regulation, some European countries (Ireland, Denmark, Germany, Sweden, Finland, Austria, Latvia, Lithuania, Netherlands, Czech Republic, and Slovakia) already introduced a national action plan to phase down dental amalgam ignoring its potential advantages [65]. Among them, dental amalgam is an excellent restorative material to be used for the caries conservative treatment in patients affected by systemic syndromes with CNS involvement [66] and in very young children [16], commonly considered uncooperative patients, which can prevent the dentist from the difficult challenge of using resins. While on the one hand, in fact, composite resins can provide a better aesthetic result; on the other hand, they need an absolutely dry and bloodless environment to guarantee a correct marginal seal. These requirements can be easily achieved with the use of the rubber dam, but this tool requires patient compliance, which, in uncooperative patients, cannot be achieved.

Even if the meta-analysis seems to demonstrate the substantial equality between dental amalgam and composite resin in terms of nephrotoxicity, it is crucial to point out that the trial sequential analysis highlighted that such meta-analysis' lack of significance must be read as “absence of evidence” rather than “evidence of absence.” This was mainly due to the moderate-high risk of bias of the included trials, to their moderate strength of evidence assessed by the GRADE, and to the low number of subjects enrolled. Unfortunately, the fact that all the enrolled studies took place before the growing containment measures against the use of amalgam demonstrates on the one hand that such measures were probably taken without correctly assessing the strength of evidence of scientific publications and on the other hand that it will be very difficult to achieve statistically significant sample numbers in the future.

Another limitation of the present review has to be pointed out: even if generally RCTs are considered “gold standard” for clinical research, they may not be long enough to assess the long-term effect of an intervention such as detection of long-term adverse effects after chronic exposure. For this reason, the design of longer RCTs for assessing various types of adverse effects linked to the use of dental amalgam is strongly suggested.

## 5. Conclusions

The statistical analyses carried out in the present systematic review demonstrate the absence of sufficient evidence to ban the use of dental amalgam for caries conservative treatments both in adults and in children. Its indisputable advantages in the treatment of very young patients and in those suffering from systemic syndromes that compromise their collaboration make it a material that can still have a fair use in dental clinical practice.

## Figures and Tables

**Table 1:** Table showing references of excluded studies after full-text evaluation with rationale for exclusion.

References	Rationale for exclusion
Barany et al. [22]	Not RCT design
Bellinger et al. [23]	Redundant publication (Bellinger et al., 2006) [24]
Berglund et al. [25]	Not RCT design
Bratel et al. [26]	Not RCT design
Herrstrom et al. [27]	Not RCT design
Leistevuo et al. [28]	Not RCT design
Levy et al. [29]	Not RCT design
Pesch et al. [30]	Not RCT design
Woods et al. [31]	Redundant publication (DeRouen et al., 2006) [32]

**Table 2:** Characteristics of the included studies.

Author and year	Methods		Participants			Intervention		Outcomes				Conclusions
	Study design	Follow-up	Country of study setting	Sample size	Mean age and gender	Materials used	Site of restoration	Renal	CNS	Social-behaviour.	Others	
Bellinger et al., 2006 [24]	RCT	5 years	USA	534	7.9 years	Dispersed phase amalgam; composite resin	Posterior teeth	Urinary Hg;	IQ	NA	Hair Hg	Urinary Hg was significantly higher level in the amalgam group; no significant differences for other outcomes.
					287 M, 247 F			Urinary Albumin	Memory			
									Visuomotor			

Bellinger et al., 2008 [35]	RCT	5 years	USA	534	7.9 years	Dispersed phase amalgam; composite resin	Posterior teeth	NA	NA	CBCL; BASC	NA	Amalgam group had significant improvement in “Total Problem Behaviour,” “Internalizing,” “Delinquent Behaviors,” “Activities,” and “Anxious/Depressed” domains (CBCL) and in “Personal adjustment” and “Emotional Symptom Index” domains (BASC).
					287 M, 247 F							

DeRouen et al., 2006 [32]	RCT	7 years	Portugal	507	10.1 years	Dental amalgam; composite resin	Posterior teeth	Urinary Hg; Urinary Albumin	Attention/concentration	NA	NA	Urinary Hg was significantly higher level in the amalgam group; no significant differences for other outcomes.
					279 M, 228 F				Memory			
									Motor/visuomotor			

Halbach et al., 2007 [33]	RCT	1.5 years	Germany	164	NR	Dental amalgam; composite resin	NR	Urinary Hg	NA	NA	Total, organic, and inorganic Hg in plasma and red cells	No statistically significant differences were found in any outcome.
					NR							

Lauterbach et al., 2008 [34]	RCT	7 years	Portugal	507	10.1 years	Dental amalgam; composite resin	Posterior teeth	NA	NHSs, NSSs, and positional tremor	NA	NA	Statistically significant higher rate of NSSs was found in the composite resin group at the second time point.
					279 M, 228 F							

Shenker et al., 2008 [36]	RCT	5 years	USA	534	7.9 years	Dispersed phase amalgam; composite resin	Posterior teeth	NA	NA	NA	WBC, T-cell, B-cell, and neutrophil and monocyte responsiveness	No statistically significant differences were found in any outcome.
					287 M, 247 F							

RCT = randomized controlled trial; M = male; F = female; Hg = mercury; IQ = intelligence quotient; NA = not available; CBCL = Child Behaviour Checklist; BASC = behaviour assessment system for children; NHS = neurological hard sign; NSS = neurological soft sign; WBC = white blood count.

**Table 3:** GRADE summary of findings for meta-analysis on urinary mercury concentration after 5 years of exposure to dental amalgam or composite resin in children.

Quality assessment and outcome: urinary mercury levels after 5 years of exposure to dental amalgam or composite resin in children						
Question: Will the use of dental amalgam for restoring dental caries in children produce an increase in urinary mercury levels?						

Number of studies according to meta-analysis	Study design	Risk of bias	Inconsistency	Indirectness	Imprecision	Publication bias
2	Clinical controlled Trials	Serious^a^	Serious^b^	Not serious	Not serious	Undetected

^a^Due to high risk of bias in one included trial. ^b^Due to heterogeneity across studies.
